# Widely-Tunable Quantum Cascade-Based Sources for the Development of Optical Gas Sensors

**DOI:** 10.3390/s20226650

**Published:** 2020-11-20

**Authors:** Virginie Zéninari, Raphaël Vallon, Laurent Bizet, Clément Jacquemin, Guillaume Aoust, Grégory Maisons, Mathieu Carras, Bertrand Parvitte

**Affiliations:** 1 Unité Mixte de Recherche 7331, Groupe de Spectrométrie Moléculaire et Atmosphérique, Centre National de la Recherche Scientifique, Université de Reims Champagne Ardenne, 51097 Reims, France; raphael.vallon@univ-reims.fr (R.V.); laurent.bizet.pro@gmail.com (L.B.); clement.jacquemin1@etudiant.univ-reims.fr (C.J.); bertrand.parvitte@univ-reims.fr (B.P.); 2 mirSense, Centre d’intégration NanoINNOV, 91120 Palaiseau, France; guillaume.aoust@mirsense.com (G.A.); gregory.maisons@mirsense.com (G.M.); mathieu.carras@mirsense.com (M.C.)

**Keywords:** quantum cascade laser, widely tunable laser sources, mid-infrared laser sources, laser spectroscopy, QCL array, external-cavity systems, intra-cavity laser absorption spectroscopy, compliance voltage technique, gas sensors

## Abstract

Spectroscopic techniques based on Distributed FeedBack (DFB) Quantum Cascade Lasers (QCL) provide good results for gas detection in the mid-infrared region in terms of sensibility and selectivity. The main limitation is the QCL relatively low tuning range (~10 cm^−1^) that prevents from monitoring complex species with broad absorption spectra in the infrared region or performing multi-gas sensing. To obtain a wider tuning range, the first solution presented in this paper consists of the use of a DFB QCL array. Tuning ranges from 1335 to 1387 cm^−1^ and from 2190 to 2220 cm^−1^ have been demonstrated. A more common technique that will be presented in a second part is to implement a Fabry–Perot QCL chip in an external-cavity (EC) system so that the laser could be tuned on its whole gain curve. The use of an EC system also allows to perform Intra-Cavity Laser Absorption Spectroscopy, where the gas sample is placed within the laser resonator. Moreover, a technique only using the QCL compliance voltage technique can be used to retrieve the spectrum of the gas inside the cavity, thus no detector outside the cavity is needed. Finally, a specific scheme using an EC coherent QCL array can be developed. All these widely-tunable Quantum Cascade-based sources can be used to demonstrate the development of optical gas sensors.

## 1. Introduction

Since the first realization of a Quantum Cascade Laser (QCL) [1], there has been a growing interest in its application to the development of optical gas sensors [2,3]. The main reason is that these semi-conductor lasers emit in the mid-infrared, where the strongest absorption lines lie for most of the absorbing gases, which is to the contrary of standard telecom diode lasers that are limited to the near-infrared, where low absorption harmonic bands lie. Various applications have emerged such as in air monitoring [4], chemical physics [5,6] or in the biomedical domain [7,8]. In this last field alone, we can cite for example a number of works dealing with cancer diagnosis [9], detection of glucose [10,11] and analysis of human respiration [12,13].

For most applications and particularly spectroscopic applications for gas detection, it is mandatory to have single frequency QCL operating devices. This tunable single-mode operation is usually reached in two ways. The first one consists in processing QCLs as Distributed FeedBack (DFB) lasers [14] and the second one by incorporating QCLs within an external cavity including a rotating diffraction grating [15].

The laser team in GSMA (Groupe de Spectrométrie Moléculaire et Atmosphérique) in Reims has a long experience in the use of DFB QCLs in spectroscopy for applications to gas detection. This team has historically developed heterodyne detection [16] in the mid-infrared range using CO_2_ lasers. The first mid-infrared heterodyne detection using QCL as local oscillator was realized in 2004 [17]. Atmospheric absorption spectra of ozone (O_3_) were recorded at 9 µm. This work was continued by other teams for Earth atmospheric studies [18,19] or astronomical ones [20,21]. DFB QCLs were also used in Reims with multipass cell in standard Tunable Diode Laser Absorption Spectroscopy (TDLAS) to demonstrate the water-vapor isotope ratio measurements in air: H_2_O^18^⁄H_2_O^16^ and HDO⁄H_2_O^16^ at 6.7 µm [22]; the simultaneous measurement of nitrous oxide (N_2_O) and methane (CH_4_) concentrations at ground level at 7.9 µm [23]; and the development of a field-deployable spectrometer using QCL technology at 4.5 μm to measure in situ N_2_O concentrations at ground level even in bad weather conditions [24]. 

In addition, the QCL power that is intrinsically higher than those of standard semi-conductor diode lasers is a key for photoacoustic gas detection [25]. Indeed the photoacoustic signal is directly proportional to the laser power. The first realization of photoacoustic spectroscopy with QCLs was demonstrated in 1999 [26]. This technique has been widely used for trace gas detection [27,28]. A recent extensive review can be found in [29]. Current works are dedicated to the development of miniaturized photoacoustic cells as the photoacoustic signal is inversely proportional to the cell volume. Two main ways are currently being explored: the Quartz-Enhanced PhotoAcoustic Spectroscopy (QEPAS) technique as for example in [30], and size-reduction of the photoacoustic cavity as for example in [31]. The advantage of QCL power for photoacoustic spectroscopy was successfully demonstrated in GSMA in [32] where the detection of methane was realized with a standard diode laser and compared with the QCL result. The high power of the QCL combined with the higher absorption coefficient in the mid-infrared had permitted to improve the detection limit of methane from the ppm to the ppb range. Then, the simultaneous detection of methane (CH_4_) and nitrous oxide (N_2_O) in atmospheric flux measurements [33] was demonstrated where a detection limit of 3 ppb of methane in air was obtained using a QCL emitting at 8 µm. Nitric oxide (NO) detection has also been performed with a photoacoustic sensor coupled with a continuous wave DFB QCL at 5.4 µm, working at cryogenic temperature [34]. 

All these GSMA realizations were obtained with continuous wave (cw) laser sources. However, still nowadays, some wavelengths can only be obtained with pulsed sources. Pulsed QCL spectrometers can be used to detect atmospheric gases in two ways [35]. The first one is the so-called interpulse technique using typically short (5–20 ns) pulses (see [36] for example). The second one called intrapulse technique uses quite long pulses (typically 500–800 ns) (see [37,38] for example). However, each of these techniques has drawbacks. Indeed, the gas absorption spectra are generally distorted due to the large influence of the pulse shape. That is why an alternative method for gas detection using pulsed QCL spectrometers was proposed in GSMA [39] and applied to the detection of ammonia (NH_3_) at 10 µm [40]. Even if one can demonstrate gas detection with pulsed QCL spectrometers, from a strictly spectroscopic point of view, spectra recorded with cw laser sources are of better quality.

The main limitation of all these experiments is the DFB QCLs relatively low tuning range (~10 cm^−1^). This drawback prevents from performing multi-gas sensing or monitoring more complex gaseous species that are characterized by broad absorption spectra in the mid-infrared region. To obtain a wider tuning range, two solutions are usually developed. The first solution consists in the use of a DFB QCL array [41]. This type of source initially developed in the Capasso group [42,43] is still under development [44,45] for the realization of optical sensors. Results for methane (CH_4_) detection using a DFB QCL array emitting from 7.2 µm to 7.5 µm are presented in Section 2.1. Another DFB QCL array associated to an arrayed waveguide grating [46] directly fixed on the chip has been developed to demonstrate the detection of carbon monoxide (CO), acetylene (C_2_H_2_) and carbon dioxide (CO_2_) with a source emitting from 4.49 µm to 4.57 µm [47]. 

A more common technique to obtain a wider tuning range that will be presented in Section 3 is to implement a Fabry–Perot QCL chip in an external-cavity (EC) system [48] so that the laser could be tuned on its whole gain curve. This technique has been demonstrated at room temperature [49] with high-power [50] for high resolution spectroscopy and chemical sensing [51]. In GSMA, the implementation of a commercial EC QCL emitting at 10.5 μm in a photoacoustic spectrometer was realized [52] and enabled measurements on a broad spectral range up to 60 cm^−1^. The tuning range of the source emitting from 10 µm to 10.5 µm demonstrates the possibility to detect small and complex molecules such as carbon dioxide (CO_2_) and butane (C_4_H_10_). Based on the same principle, an EC QCL spectrometer was designed in the lab at 7.5 μm. The QCL and its anti-reflection coating were specially developed for this application [53]. A continuous wave emission from 1293 up to 1350 cm^−1^ was demonstrated, and measurements on acetone (C_3_H_6_O) and phosphoryl chloride (POCl_3_) were realized. 

The use of an EC system also allows to perform Intra-Cavity Laser Absorption Spectroscopy (ICLAS). This technique consists of placing the gas sample within the laser resonator. In this case, absorption lines of the sample influence the spectrum during many round trips. The cavity output light can be detected to perform spectroscopy. This method, already well known in the visible [54,55,56] and the near infrared [57,58,59,60], is developing in the mid-infrared [61,62]. With a carefully optimized system, the effective pathlength can reach hundreds of kilometers, thus making it an appropriate technique for low absorption gases detection. Moreover, a technique using the QCL compliance voltage may be developed to retrieve the spectrum of the gas inside the cavity. In this way, no detector outside the cavity is needed as the cavity is the light source and the detector at the same time [63]. Preliminary results using this technique in GSMA and demonstrating the detection of methane (CH_4_) and water vapor (H_2_O) at 7.6 µm will be presented.

Finally, another specific scheme combining EC systems and QCL array will be anew briefly presented in Section 4. This new design is based on coherent quantum cascade laser micro-stripe arrays [64]. Phase-locking is provided by evanescent coupling between adjacent stripes, thus the far-field pattern shows a dual-lobe emission. This a priori drawback has been converted to a force by developing an EC system with this source, and acetone spectra have been recorded at 8.2 µm [65]. Another team has used the same design in [66].

## 2. Distributed FeedBack (DFB) Quantum Cascade Laser (QCL) Array

To obtain a wide tuning range, the first solution consists in the use of a DFB QCL array [41]. A first system of DFB QCL array emitting from 7.2 µm to 7.5 µm has been developed in GSMA with the source from mirSense and will be presented in Section 2.1. This source has been used to demonstrate methane (CH_4_) detection. However, the main drawback of this monolithic source is the lateral shift of the beam with operating laser switching. A moveable lens and a parabolic mirror for the detection system can be used to compensate the shift. Moreover, it is a major drawback when using systems that are sensitive to alignment such as multipass cells. The beam lateral shift due to the numerous outputs of the array may be compensated by using a diffraction grating to group the wavelengths [67] or by the fabrication of integrated waveguides collecting beams in a single output [68]. In Section 2.2, an intermediate solution between these two methods demonstrated in GSMA will be anew presented. The DFB QCL array is combined with an Arrayed Waveguide Grating (AWG), usually called multiplexer, directly fixed on the chip. Thus, the system (QCLA+AWG) is not strictly monolithic but it is no more mandatory to realign the system when switching between lasers. The developed system is used to demonstrate the detection of carbon monoxide (CO), acetylene (C_2_H_2_) and carbon dioxide (CO_2_) with a source emitting at 4.5 µm [47].

### 2.1. DFB QCL Array without Multiplexer

**Figure 1:**
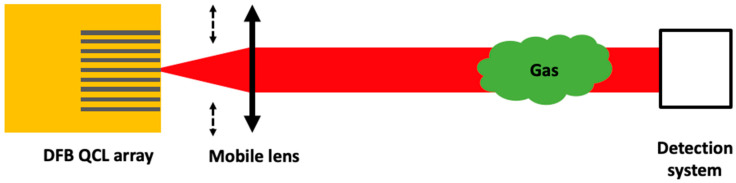
Schematic of a DFB QCL array source for gas detection. Grey lines in the yellow square indicate the lasers in the array. A mobile lens following the dashed arrows must be moved to adapt to the output of the chosen laser.

The principle of the DFB QCL array without multiplexer and its application to gas detection is presented in Figure 1. 

**Figure 2:**
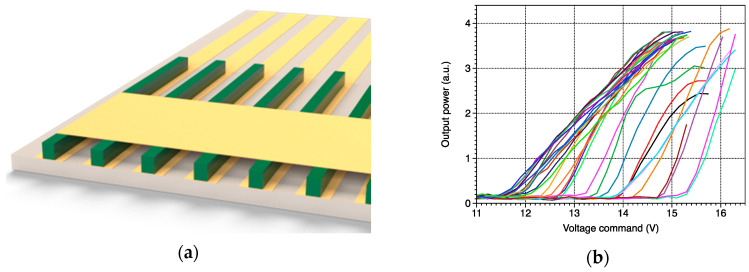
(**a**) Schematics of the DFB QCL array. The electrical contact (in yellow) of the DFB QCLs (in green) is given by a gold common terminal and by gold individual tracks under the lasers; (**b**) Power of the 34 QCLs of the array vs. voltage command. Supply conditions of all pulsed lasers are: temperature 20 °C, pulse duration 100 ns, repetition rate 100 kHz, peak current 1.3 A.

A room temperature DFB QCL array from mirSense is mounted onto a homemade water cooling with single stage Peltier element. Each laser has an approximate 20 µm^2^ emission surface and the lasers are periodically spaced by about 100 µm. The DFB grating of each QCL is slightly different in order to have different wavenumbers. The electrical contact is given by a gold common terminal and by gold individual tracks under the lasers (see Figure 2a). A QCL pulser switching unit (LDD100, Alpes Lasers SA, St-Blaise, Switzerland) converts the voltage control into current pulses. The latest are transported to the array by a low impedance cable to avoid any signal deformation. The duration and the frequency of the pulses are determined by a pulse generator (BNC565, Berkeley Nucleonics Corporation, San Rafael, CA, USA). The array is composed of 34 DFB QCLs that are working in quasi-continuous regime with an optical power of about 1 mW. Figure 2b shows the laser output power evolution of each QCL from the array with the voltage command. They all have the same tendency but with different thresholds and maximum powers. No correlation was observed between the emitted wavenumber and the threshold value. These disparities are due to imperfections during the array fabrication. For this particular source, each DFB QCL is addressed by a single three-axis motorized tungsten probe with 0.3 micrometer accuracy and a metallic stripe on the common gold terminal. The probe movement is automated and controlled by a Labview program. 

**Figure 3:**
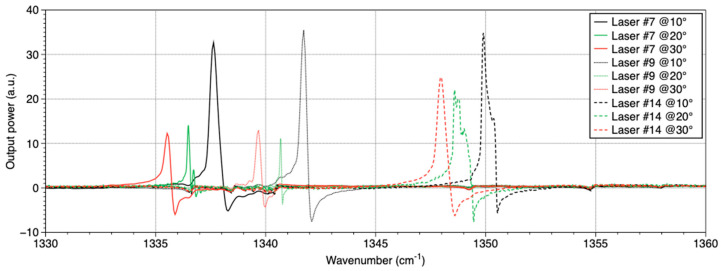
Emission spectra of the QCLs #7, #9, and #14 from the DFB QCL array for three temperatures.

**Figure 4:**
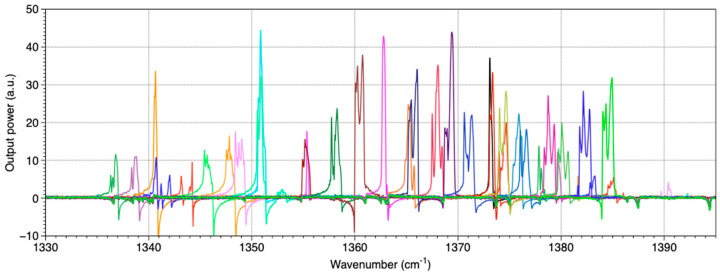
Emission spectra of all the QCLs from the DFB QCL array for one temperature (20 °C).

A spectral variation is obtained by varying the array temperature, which is controlled by a TEC2000 from Thorlabs (Newton, NJ, USA) associated with a Peltier element. The wavenumber measurement is realized with a Fourier Transform InfraRed spectrometer (FT/IR-6300 from Jasco, Easton, MD, USA) with a minimal accuracy of 0.015 cm^−1^. Figure 3 presents the emission spectra in pulsed mode from three DFB lasers of the array recorded for three different temperatures. Each laser covers a spectral range of about 2–3 cm^−1^ by tuning the temperature from 10 to 30 °C. A wide continuous spectral range can be obtained as each spectral range overlaps other ones. This result is presented in Figure 4. 

A total range of more than 50 cm^−1^ from 1335 to 1387 cm^−1^ can be achieved. Note that the FTIR used to record those spectra is not time-resolved and cannot be synchronized with the laser emission. Thus, the recorded spectra may be distorted and cannot be used to demonstrate single-mode emission but only to evaluate the emission wavenumber. 

As presented in Figure 1, the DFB QCL array source developed for gas detection has to be used with a 4 mm focal length lens mounted on a motorized translation (M-112.1DG from Physik Instrumente, Karlsruhe, Germany) in order to collimate the beam of each laser of the array. The gas of interest is methane filled in a 15-cm-long cell at ambient temperature and at 0.1 bar pressure. As the beam moves with each laser change, the detection system (a HgCdTe detector at liquid nitrogen temperature) is placed after a parabolic mirror that compensates the beam shift and focalizes the beam on the detector. 

**Figure 5:**
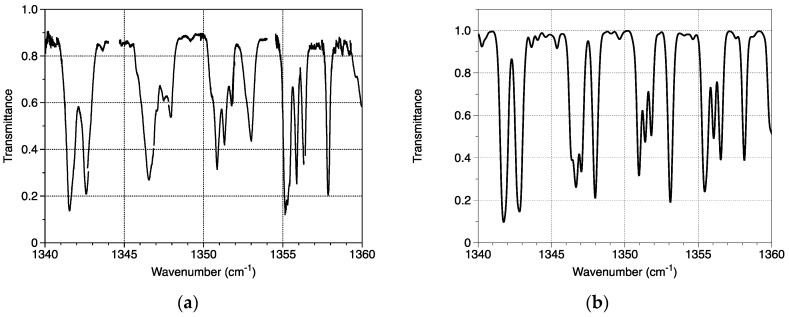
(**a**) Transmission spectra of the DFB QCL array through the methane gas cell and (**b**) transmittance calculated from HITRAN data in the same conditions than the experience.

Each laser operates in quasi-continuous regime with 100 ns pulses and a period of 10 µs. Signals are recorded during a 90 s long temperature ramp between 10 °C and 30 °C. The power transmitted through the gas cell and the chip temperature are synchronously recorded by an oscilloscope (Waverunner 104Xi from Teledyne Lecroy, Chestnut Ridge, NY, USA). The gas transmission spectrum is obtained from two successive records with and without gas. The conversion from temperature to wavenumber is obtained assuming that its variation is linear for a DFB laser. The result of the measurement on a part of the whole wavenumber range is presented in Figure 5a. Figure 5b shows the calculated transmission from HITRAN data [69] in the same experimental conditions when adding an apparatus function of 0.25 cm^−1^ to be close to the one of the spectroscopic system. One can remark a good agreement on the wavenumber data and the quite good agreement with the absorption depths. 

This first system of DFB QCL array emitting from 7.2 µm to 7.5 µm has been used to demonstrate the possible detection of methane. This laser source is a first prototype and emits only in the pulsed mode with a low power. Thus, at the moment, this kind of source cannot be competitive with current existing optical sensors using high-power standard DFB QCLs. However, the next prototypes that would emit in cw mode with a higher power should be competitive either for multi-species gaseous molecules such as methane and/or for the detection of complex species with broad absorption spectra in the mid-infrared region thanks to its wide emission range. For example, in the demonstrated spectral range and using the photoacoustic spectrometer demonstrated in [33] in combination with this type of source emitting 20 mW in continuous mode, one could be able to demonstrate the simultaneous detection of methane (CH_4_), sulphur dioxide (SO_2_), and water vapor (H_2_O) with 5 ppb, 2 ppb and 20 ppb respective detection limits. Moreover, this emission range is totally suitable for the detection of acetone (C_3_H_6_O) and propyne (C_3_H_4_). The detection limits that could be achieved with the photoacoustic spectrometer [33] are 5 ppb and 120 ppb, respectively. Finally, this source should also be used for the detection of other complex species with very good detection limits: 60 ppb of acetonitrile (CH_3_CN), 70 ppb of acrolein (C_3_H_4_O), 40 ppb of propane (C_3_H_6_), and 1 ppb of nitrobenzene (C_6_H_5_NO_2_).

### 2.2. DFB QCL Array with Multiplexer

**Figure 6:**
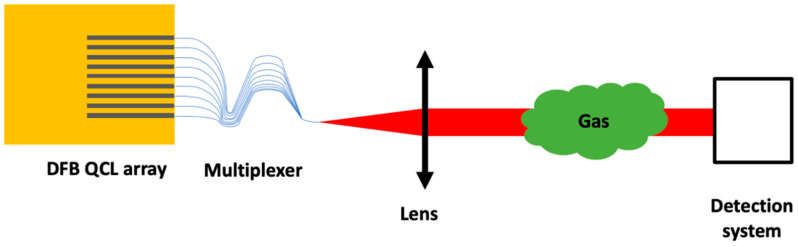
Schematic of a DFB QCL array source with multiplexer for gas detection.

As previously mentioned, the beam lateral shift with laser changing is the main drawback of the monolithic source presented in Section 2.1. It imposes the use of a moveable lens and a parabolic mirror for the detection system. Moreover, its use with systems that are sensitive to alignment such as multipass cells becomes far more complicated. This drawback is avoided with the system presented in this section. The principle of the DFB QCL array with multiplexer and its application to gas detection realized in GSMA with a source from mirSense is presented in Figure 6. 

A 2190–2220 cm^−1^ spectral range covered by the chip has been reached. Carbon monoxide (CO) spectra were recorded with a single-path 51-cm-long cell. Flame gas mixture spectra were also recorded where CO and acetylene (C_2_H_2_) lines may be identified. Finally, in order to demonstrate the efficiency of the multiplexer, the developed system was combined with a multi-pass 5.1-m-long White cell to demonstrate detection of carbon dioxide (CO_2_). More details on this experiment can be found in [47].

## 3. External-Cavity Quantum Cascade Laser (EC QCL)

### 3.1. Standard EC QCL Systems and Intra-Cavity Laser Absorption Spectroscopy

**Figure 7:**
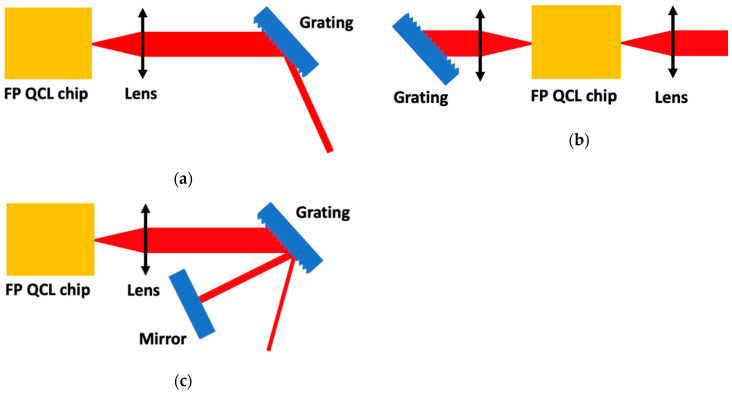
Schematic of Fabry–Pérot Quantum Cascade Laser chip source in an external-cavity system in (**a**) front facet Littrow configuration, (**b**) back facet Littrow configuration and (**c**) front facet Littman configuration.

**Figure 8:**
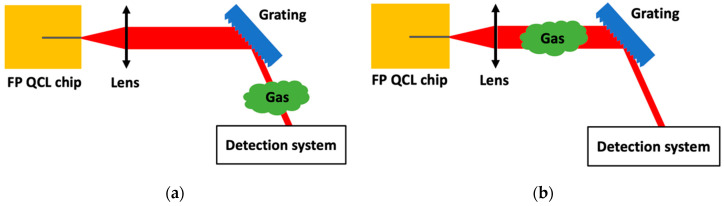
(**a**) Schematic of a standard Fabry–Pérot Quantum Cascade Laser chip source in an external-cavity system for gas detection in front facet Littrow configuration and (**b**) the same set-up used to perform Intra-Cavity Laser Absorption Spectroscopy.

A more common technique to obtain a wide tuning range is to implement a Fabry–Perot QCL chip in an external-cavity (EC) system so that the laser could be tuned on its whole gain curve [48]. The most standard solutions for EC-systems are presented in Figure 7. Figure 8a presents the front facet Littrow configuration (Figure 7a) applied to gas detection.

In GSMA, the implementation of a commercial EC QCL emitting at 10.5 μm in a photoacoustic spectrometer was realized and enables measurements on broad spectral range up to 60 cm^−1^. The tuning range of the source emitting from 10 µm to 10.5 µm demonstrates the possibility to detect small and complex molecules such as carbon dioxide (CO_2_) and butane (C_4_H_10_) [52]. Based on the same principle, an EC QCL spectrometer was designed in the lab at 7.5 μm. Two methods were used for the rotation of the grating: step by step and continuous rotation. The continuous rotation method provided good results for a shorter acquisition time. A continuous wave emission from 1293 up to 1350 cm^−1^ was demonstrated, and measurements on acetone (C_3_H_6_O) and phosphoryl chloride (POCl_3_) were realized [53]. The use of an EC system also allows to perform Intra-Cavity Laser Absorption Spectroscopy (ICLAS) where the gas sample is placed within the laser resonator (see Figure 8b). The ICLAS was developed at the beginning of the 70s [70]. This method, already well known in the visible and the near infrared, is developing in the mid-infrared [61,62]. Absorption lines influence the spectrum during many round trips. The cavity output light can be detected to perform spectroscopy. With a carefully optimized system, the effective pathlength can reach hundreds of kilometers, thus making it an appropriate technique for low absorption gases detection. 

### 3.2. EC QCL—Voltage Intracavity Sensing

**Figure 9:**
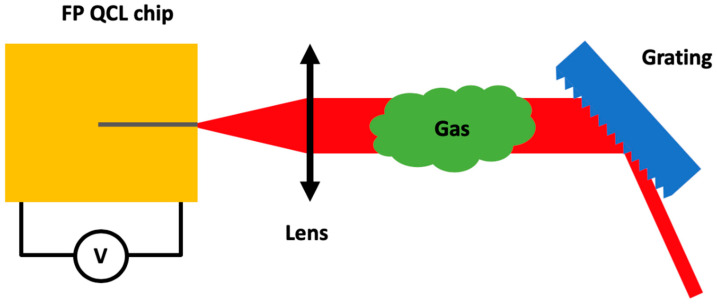
Schematic of an External Cavity Quantum Cascade Laser—Voltage Intracavity Sensing for gas detection in front facet Littrow configuration. Note that, in this case, no detection system is needed as the cavity is the light source and the detector at the same time.

In addition to the ICLAS technique, a technique using the QCL compliance voltage can be used to retrieve the spectrum of the gas inside the cavity, thus no detector outside the cavity is needed [63]. This technique is based on the fact that the compliance voltage of a semi-conductor laser is sensitive to optical feedback. For EC QCLs, the cavity losses can be changed by introducing absorbing materials such as gases into the laser cavity. The principle of the EC QCL—Voltage Intracavity Sensing and its application to gas detection is presented in Figure 9.

In order to test this technique in GSMA, a previously existing EC QCL mounted in back facet Littrow configuration (Figure 7b) was used with a QCL device designed for operation at a center wavelength of 4.5 μm. The external cavity length of 5 cm used a diffraction grating blazed for 4.5 μm with an angle of 44.8°. Although it was possible to demonstrate a wide emission range from 2050 to 2190 cm^−1^, a lot of QCL mod-hops appear every 0.31 cm^−1^, which is logical for a QCL length of 0.5 cm and a refractive index of 3.27. Moreover, as there was some problems of mechanical instabilities with the rotation platform of the grating, it was never possible to record gas spectra with this system.

Five QCL chips were tested with a front facet Littrow configuration (Figure 7a) in order to choose the more powerful chip in cw mode with the wider wavelength range. The best chip has a cw max power of 16 mW and emits in pulsed operating mode from 1283 to 1384 cm^−1^. A second EC QCL mounted in Littman configuration (Figure 7c) was assembled using the best QCL device designed for operation at a center wavelength of 7.5 μm. The Littman configuration was chosen in order to have a fixed beam at the output of the cavity and had an external cavity length of 38 cm. This quite big value was chosen in order to have sufficient place to insert a 20-cm-long gas cell in the set-up. The EC QCL used a diffraction grating blazed for 8 μm with an angle of 36.5°. The first-order diffraction from the grating was directed to a galvanometer-mounted tuning mirror (model 6220H from Cambridge Technology, Bedford, MA, USA), which selected the operation wavelength of the EC QCL. 

The QCL chip is placed inside a modified Laser Laboratory Housing (LLH) adapted for mirSense chips. The EC QCL can be operated in both pulsed and continuous-wave configurations. For pulsed operation, a Digital Delay/Pulse generator from Berkeley Nucleonics Corporation commands a pulser switching unit (LDD100 from Alpes Lasers SA, St-Blaise, Switzerland) that generates a current pulse transported to the array via a low-impedance flat cable. For cw operation, a stabilized current supplier LDX-3232 from ILX Lightwave (Bozeman, MT, USA) with a 4 A maximal current is used. The QCL device temperature is controlled by a LDT-5980 from Newport (Irvine, CA, USA) associated with a PT100 and a Peltier element.

**Figure 10:**
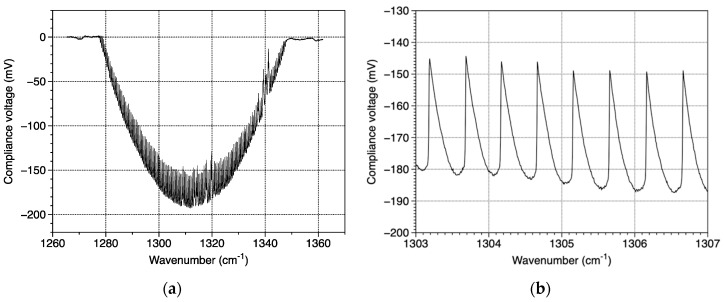
(**a**) QCL device compliance voltage when varying the mirror angle ±3° and (**b**) a zoom on Figure 10a around 1305 cm^−1^.

The compliance voltage of the QCL device was measured via a FPGA PXI-7841 card from National Instruments (Austin, TX, USA) that can record up to 2 × 10^5^ samples/s. The compliance voltage variation of the EC QCL across a mirror movement of ±3° corresponding to a wavenumber variation of about 100 cm^−1^ is presented in Figure 10a. Such a figure with a U form is called mark. Figure 10b presents a detail of the mark of Figure 10a. The magnitude of the compliance voltage is negative due to the polarity of the QCL device. Figure 10a shows two different types of peaks inside the mark. The first one that is zoomed in Figure 10b corresponds to QCL mod-hops. The interval between two peaks equals 0.5 cm^−1^, which corresponds to the free spectral range (FSR) of the QCL chip. Notwithstanding these peaks, one can remark other peaks around 1320 cm^−1^ and 1340 cm^−1^. These peaks correspond to water vapor absorption lines because of the presence of water vapor in the air of the cavity.

**Figure 11:**
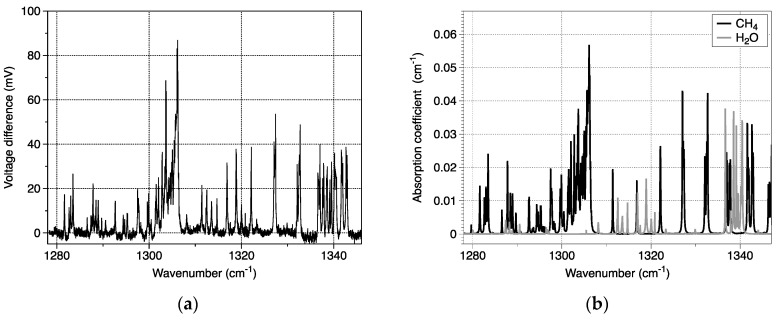
(**a**) Experimental spectra and (**b**) absorption coefficient calculated from HITRAN data assuming 0.3% of methane and 3% of water vapor in ambient air at atmospheric pressure.

In order to suppress mod-hops on the gas spectra, a new acquisition method has been developed [71]. It is based on a simultaneous wavenumber double scanning. A broad scanning realized by the mirror covers the whole spectral range of the laser. Another finer scanning is operated by a current ramp so as to cover the FSR of the QCL. The current ramp is much slower than the mirror ramp. Thus, each recorded mark is spectrally shifted to another one. Several processing methods have been tested [71] on two records, with and without gas inside the cell. The best one allows to demonstrate preliminary results on the detection of methane (CH_4_) and water vapor (H_2_O) at 7.6 µm. Figure 11a presents the voltage difference between the two records with and without gas. The gas inside the cavity corresponds to 0.3% of methane in ambient air at atmospheric pressure. One can also estimate that ambient air contains approximately 3% of water vapor. Figure 11b shows the corresponding absorption coefficient in the experimental conditions. Once again, one can observe the good agreement between experimental and calculated spectra in terms of absorption positions and depths.

## 4. External Cavity Coherent Quantum Cascade Laser Array 

Finally, another specific scheme combining EC systems and QCL array is anew briefly presented in this part. It is based on coherent quantum cascade laser micro-stripes (µ-stripes) array [64]. The main goal is to obtain a very powerful source. This coherent µ-stripes array configuration was proposed to solve both the problems of beam quality and thermal dissipation of wide active region InP-based QCLs. Phase-locking is provided by evanescent coupling between adjacent stripes and leads to the creation of a so-called supermode. In the proposed configuration, the far-field intensity pattern for this supermode is a dual-lobe emission. The spacing of the µ-stripes greatly increases the thermal dissipation of the device and thus reduces the laser heating when operating at high power.

**Figure 12:**
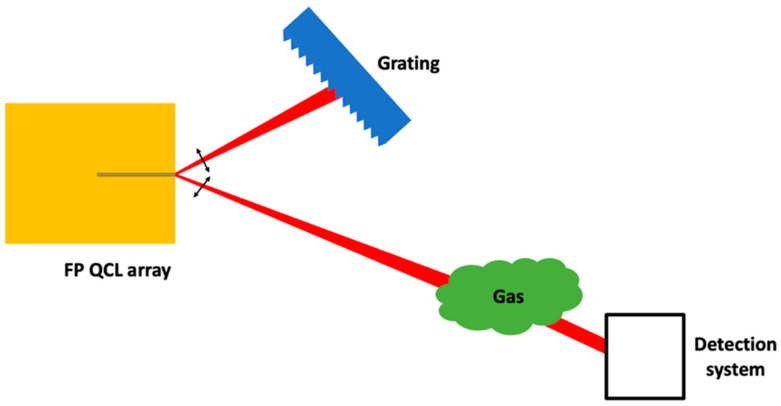
Schematic of an External Cavity Coherent Quantum Cascade Laser Array for gas detection.

The source emitting at 8.2 µm is formed of 4 µ stripes 2 µm width and 8 µm spacing. The a priori drawback of dual-lobe emission (with an angular separation of almost 45°) has been converted into a force by developing an EC system with this source. One lobe is used as an output beam while the optical feedback is obtained on the other lobe using a diffraction grating in Littrow configuration. The schematic of this configuration and its application to gas detection is presented in Figure 12. The possibility to tune up to 40 cm^−1^ of the emitted wavelength was demonstrated [65]. This source was implemented with a cell containing acetone. The obtained experimental spectrum is in good agreement with a FTIR spectrum and a spectrum from PNNL database [72]. To our knowledge, this was the first realization of an external cavity with a coherent laser array in the mid-infrared. More details on this experiment can be found in [65].

## 5. Conclusions

The laser team in GSMA in Reims has a long experience in the use of Distributed FeedBack Quantum Cascade Lasers in spectroscopy for applications to gas detection. The main limitation of all these experiments is the DFB QCLs’ relatively low tuning range (~10 cm^−1^) that prevents from monitoring complex species with broad absorption spectra in the infrared region or performing multi-gas sensing. 

To obtain a wider tuning range, the first solution that has been presented in Section 2 of this paper consists in the use of a DFB QCL array. Methane (CH_4_) detection using a DFB QCL array emitting from 7.2 µm to 7.5 µm has been demonstrated. In a second time, another DFB QCL array associated to a multiplexer directly fixed on the chip has been presented to demonstrate the detection of carbon monoxide (CO), acetylene (C_2_H_2_) and carbon dioxide (CO_2_), with a source emitting around 4.5 µm. As a first proof of concept, to our knowledge, the prototypes demonstrated in this work show a lot of possibilities for multi-gas sensing and for monitoring complex species with broad absorption spectra in the infrared region. One of the next step for these QC-based sources will be to obtain continuous wave functioning, because spectra that are recorded with pulsed sources remain of lower quality than those recorded with cw sources. Another step will consist in the improvement of the covered spectral range, first up to 100 cm^−1^ then up to 200 cm^−1^.

A more common technique to obtain a wider tuning range that has been presented in Section 3 is to implement a Fabry–Perot QCL chip in an external-cavity (EC) system so that the laser could be tuned on its whole gain curve. The use of an EC system also allows to perform Intra-Cavity Laser Absorption Spectroscopy (ICLAS), where the gas sample is placed within the laser resonator. Moreover, a technique using the QCL compliance voltage can be used to retrieve the spectrum of the gas inside the cavity, thus no detector outside the cavity is needed. Preliminary results demonstrating the detection of methane (CH_4_) and water vapor (H_2_O) at 7.5 µm have been presented. Finally, another specific scheme combining EC systems and QCL array has been anew presented in Section 4. For this last system, the main next step will be to realize the same type of source but emitting in cw mode. On the opposite of pulsed emission presented in Section 2.1, cw emission is more simple to implement. Moreover, the quality of the spectra obtained is much better and helps in obtaining good spectroscopic measurements. In addition, in this case, the average power increases, which makes cw sources ideal for photoacoustic spectroscopy, where the signal is directly proportional to the laser power.

For EC systems, the overall tuning range is usually limited by two main parameters. The first one corresponds to the width of the gain curve of the FP QCL chip. This value is defined by the design of the chip. The second one is linked to the reflection at the output of the QCL FP chip. This parameter must be minimized in such a coupled cavities system in order to help the feedback from the diffraction grating. This can be achieved through the deposition of an anti-reflection (AR) coating on the front face of the QCL chip. Thus, the development of new AR coatings and of a broadband gain middle QCL chip are key issues for external cavity systems and will consist in the next steps of these works. For the particular compliance voltage system, next improvements will consist in making the set-up stability better, to shorten the cavity length and to test the limits of the developed system by the measurement of voltage evolution with gas concentration. A more long-term objective consists in the development of real time sub-ppm detection.
